# MicroRNA Profile of Human Bone Marrow Mesenchymal Stem Cells during Hepatic Differentiation and Therapy

**DOI:** 10.7150/ijms.67639

**Published:** 2022-01-01

**Authors:** Jing Jiang, Jiaojiao Xin, Wenchao Ding, Dongyan Shi, Suwan Sun, Beibei Guo, Xingping Zhou, Chufan Zheng, Jun Li

**Affiliations:** 1 State Key Laboratory for Diagnosis and Treatment of Infectious Diseases, Collaborative Innovation Center for Diagnosis and Treatment of Infectious Diseases, The First Affiliated Hospital, Zhejiang University School of Medicine, 79 Qingchun Rd., Hangzhou, 310003, China.; 2 Hangzhou No.14 High School, 580 Fengqi Rd, Gongshu District, Hangzhou, 310006, China.

**Keywords:** microRNA, human bone marrow mesenchymal stem cells, hepatic differentiation, fulminant hepatic failure

## Abstract

**Background and Aims:** MicroRNAs (miRNAs) play important roles in hepatocyte differentiation from human bone marrow mesenchymal stem cells (hBMSCs) and the therapeutic application *in vivo*. However, the mechanisms of miRNA regulation are still unknown. This study aimed to profile the miRNA basis for improving the function of hBMSC-differentiated hepatocyte-like cells (hBMSC-Heps).

**Methods:** Characteristic miRNAs of hBMSC-Heps were identified by transcriptome sequencing and validated by quantitative real-time polymerase chain reaction (qRT-PCR). An *in vivo* hBMSC transplantation model was used to assess the regulatory effects of miRNAs on liver regeneration during hBMSC therapy in pigs with fulminant hepatic failure (FHF). The biological functions of significant miRNA molecules were confirmed by transfection of miRNA activators or inhibitors into hBMSCs during hepatogenic differentiation.

**Results:** The transcriptome of hBMSC-Heps showed characteristics distinct from those of undifferentiated hBMSCs. A total of 77 miRNAs were significantly differentially expressed in hBMSC-Heps at day 10 and day 20 after hBMSC differentiation that were directly related to the functions of hepatocytes. Among the top 10 significantly differentially expressed and the top 10 most abundant miRNAs, nine miRNAs that exhibited a pattern of gradual change were chosen for further analysis. The expression of nine miRNAs was confirmed by qRT-PCR *in vitro* and showed the same changing trends *in vivo* in an hBMSC transplantation model in pigs. Functional experiments with these miRNAs showed that activators of hsa-miR-26b-5p and hsa-miR-148a-3p and an inhibitor of hsa-miR-423-3p were sufficient to improve the differentiation of hBMSCs into hepatocyte-like cells.

**Conclusions:** Transcriptome profiles of miRNA revealed the basis of the differentiation and development of hBMSC-Heps. Manipulation of three miRNAs (hsa-miR-26b-5p, hsa-miR-148a-3p and hsa-miR-423-3p) significantly improved hepatocyte generation and liver regeneration, indicating the potential of these miRNAs for future clinical applications.

## Introduction

Fulminant hepatic failure (FHF) is a lethal syndrome that involves the acute deterioration of hepatic and extrahepatic organs which associated with high mortality 1, 2. Liver transplantation is the only option for saving patients with FHF, but transplantation is limited by the shortage of donors and by contraindications 3. Among several strategies developed to overcome this situation, stem cell-based therapies hold substantial promise 4, 5, 6. The hepatic differentiation ability of stem cells has been characterized *in vivo* and *in vitro* in many previous studies from our laboratory and others, and the results suggest that this strategy is an excellent candidate for the treatment of FHF and end-stage liver disease 7, 8, 9, 10. Our previous study demonstrated that transplantation of human bone marrow mesenchymal stem cells (hBMSCs) can rescue FHF pigs through stem cells proliferation and differentiation into hepatocytes 9. As an alternative, hBMSC-derived hepatocyte-like cells (hBMSC-Heps) serve as a potential source for hepatocyte transplantation and bioartificial liver systems 7, 11. Although the ability of hBMSCs to differentiate into hepatocytes *in vitro* and *in vivo* has been widely recognized, most studies have focused on the therapeutic promise of stem cells; the mechanism of hBMSC differentiation and the performance of hBMSC-Heps are still unclear. A systematic evaluation of hBMSC-Heps is needed to pave the way for their future clinical application by revealing their distinct functions and assessing potential risks.

Recently, microRNAs (miRNAs) have attracted great attention because they regulate up to 30% of human genes, and their influence underlies almost every aspect of biological processes at both the transcriptional and posttranscriptional levels. miRNAs are the most abundant class of small endogenous noncoding regulatory RNA molecules and generally function by inhibiting gene expression or silencing genes through destabilization and degradation of their target mRNA 12, 13. Most miRNAs are evolutionarily conserved but exhibit developmental timing and tissue specificity 14, 15. Many studies have demonstrated that miRNAs play an important role in several physiological and pathological processes in the liver 16 and have profound effects on biological processes such as cell development, differentiation and proliferation. The expression of specific miRNAs at different stages during early human liver development 17, 18 and during hepatic regeneration following partial hepatectomy 19 has been well described. As key regulators controlling cell fate and tissue development, miRNAs might serve as efficient molecular switches that control the fate of stem cells 16, 20. Studies have also addressed the function of miRNAs in liver development, showing that the expression of miRNAs plays a decisive role in stem cells differentiation into hepatocytes or cholangiocytes 21 and promotes hepatic differentiation 22, 23. Recently, large-scale miRNA expression profiling of stem cells has become a major focus of research efforts. Studies have revealed some specific expression signatures of hepatic differentiation through miRNA arrays of human embryonic stem cells 24 and umbilical cord mesenchymal-derived stem cells 25, but hBMSCs have not been investigated in this way. However, these studies have been limited to comparing the undifferentiated and differentiated states of stem cells *in vitro*. The differences between hBMSC-Heps and primary human hepatocytes (PHHs) and the details of the differentiation process *in vitro* and *in vivo* are still unclear.

In this study, the miRNA basis of hBMSC-derived hepatocyte-like cells was compared with that of PHHs and validated *in vivo* with an hBMSC transplantation model in FHF pigs. Overall, we found three miRNA molecules that can improve hepatic differentiation and generate more functional hBMSC-Heps for future treatment of various liver diseases.

## Methods

### Cell isolation, culture and phenotypic identification

HBMSCs were isolated from bone marrow that was collected from the iliac crest of 4 healthy volunteers whom given informed written consent. The hBMSCs derived from each volunteer were isolated as previously described 26. Phenotypic identification of cultured hBMSCs was performed by standard flow cytometry. hBMSCs (passage 3-7, 1×10^6^ cells) were incubated for 60 min with PE- or FITC-conjugated mouse monoclonal antibodies, namely, anti-human CD29, anti-human CD45 (Abcam, Cambridge, UK), anti-human CD34 (Santa Cruz Biotechnology, Santa Cruz, CA, USA), or anti-human CD90 (BD Biosciences, San Jose, CA, USA), or with an immunoglobulin isotype as a negative control. The cells were analyzed with a FACS Calibur system (FC500, Beckman Coulter, Fullerton, CA, USA) after washing. PHHs were isolated using a four-step collagenase perfusion method as previously described 27. Briefly, the liver segments were perfused by the extracorporeal circulating perfusion apparatus with four different buffer solutions supplemented with 0.37 mg/ml EDTA, 0.5% dispase, 0.05% collagenase type IV (Gibco BRL, Grand Island, NY), and 40 µg/ml DNase I (Sigma, St. Louis, MO). The remnants were dispersed mechanically with a scalpel blade and filtered sequentially through a 100 µm nylon mesh. The isolated cells were harvested and centrifuged at 4 °C and 50 g for 3 min.

### Hepatic differentiation of hBMSCs

Hepatic differentiation of harvested hBMSCs was performed according to the methods described in our previous study 8, 9. Briefly, hBMSCs (third passage, 1×10^6^ cells) were cultured with hepatocyte differentiation basal medium containing 20 ng/ml human hepatocyte growth factor (hHGF); 20 µg/ml dexamethasone; 50 mg/ml insulin, transferrin, and selenium (ITS) premix; and 100 U/ml penicillin and 100 µg/ml streptomycin for 2 weeks. After that, the cells were cultured in another medium including 20 ng/ml oncostatin M (OSM), 20 µg/ml dexamethasone, 50 mg/ml ITS premix, 100 U/ml penicillin and 100 µg/ml streptomycin for an additional week. Hepatic functions were characterized prior to hBMSC differentiation and then at 10 and 20 days after differentiation. The expression of liver-specific genes (amplified by the primer sequences listed in Table S1) was analyzed by qRT-PCR, albumin (ALB) expression was measured by immunostaining, and glycogen synthesis was analyzed by periodic acid-Schiff (PAS) staining system (Sigma, St. Louis, MO) using methods described previously 8. Briefly, the fixed specimen slides immersed in Schiff's reagent for 15 min at room temperature and rinsed in running tap water for 5 min. After that, the slides were counterstained in Hematoxylin solution for 90 s, washed in running tap water for 15-30 s, dried and examined microscopically.

### hBMSC transplantation in an FHF pig model

An in-house translational model was used as previously described 9. Briefly, an FHF pig model was induced with D-galactosamine (1.5 g/kg body weight) via jugular vein catheterization of male Chinese miniature experimental pigs (Taihe Biotechnology, Jiangsu, China) weighing 8-10 kg and aged 2.5 months. The FHF pigs were randomized into two groups: an hBMSC intraportal transplantation group that received a transfusion of hBMSCs (3×10^6^/kg, suspended in 10 ml of normal saline) via the intrahepatic portal vein under B-ultrasound guidance and a control group that underwent a sham procedure with an equal volume of normal saline without cells. All transplantation operations were performed by the same B-ultrasound expert with more than 5 years of experience.

### RNA isolation and miRNA sequencing (miRNA-seq)

Total RNA was extracted and purified from cells with TRIzol reagent (Invitrogen, CA, USA) following the manufacturer's instructions. The cell samples included undifferentiated hBMSCs (day 0; D0 group) and hBMSC-Heps at 10 days (D10 group) and 20 days (D20 group) after hepatic differentiation (n=4/group), with freshly isolated PHHs as positive controls (n=3). A sequencing library was prepared using the Illumina TruSeq™ Small RNA Sample Preparation Guide; the steps included adapter ligation, reverse transcription, PCR amplification, and gel purification. Deep sequencing of small RNAs was performed using an Illumina HiSeq 2000 sequencing system (Illumina, San Diego, CA, USA). The average number of sequencing reads was approximately 10 million per RNA sample.

### Bioinformatics analysis of sequencing data

Prior to further analysis, the sequencing reads were preprocessed by cutting the 3' adapter sequence using the cut-adapt program. After clipping, the sequence reads shorter than 18 nucleotides were removed. Furthermore, a dataset that consisted of unique sequences with their associated read counts was obtained by removing the identical sequences and recording the remaining reads. To improve the efficiency and reliability of the mapping steps, the singleton reads were not included in subsequent analyses. The obtained sequences were aligned to the human genome (hg19) downloaded from the University of California, Santa Cruz (UCSC) genome database with the program miRDeep2 and mapped against miRNA precursor sequences from miRBase release v21 28. For each sample, the read counts of the miRNAs were calculated and recorded.

### Validation of miRNAs *in vitro* and *in vivo*

Liver tissues were collected from the experimental pigs in the normal group (PN), FHF pigs in control group at day 3 after D-galactosamine induction (PC-D3), and pigs in hBMSCs transplantation group at week 3 and week 5 (PT-3W/PT-5W). To determine the effect of the transplanted hBMSCs on liver regeneration, liver tissues were analyzed using Hematoxylin and eosin (H&E) staining and immunohistochemistry with the human hepatocyte-specific marker ALB (Bethyl, Texas, USA) and an HSA (Abcam, Cambridge, UK). For H&E staining, the liver tissue section was heat-fixed at 60 °C for 1 hour and stained with H&E as described previously 9.

Selected genes, including liver-specific genes and significantly differentially expressed miRNAs from the miRNA-seq experiments, were further validated by qRT-PCR using an ABI 7500HT instrument (Applied Biosystems, Waltham, MA, USA), as described previously 29. The qRT-PCR was performed with a two-step protocol following the manufacturer's instructions. Briefly, total RNA was reverse transcribed with a miRNA-specific primer, and then qRT-PCR was performed with TaqMan probes (Invitrogen, CA, USA). The target genes were assayed in triplicate on each plate. Significantly differentially expressed miRNAs in the above cell and tissue samples were selected for qRT-PCR validation.

### Target prediction of differentially expressed miRNAs and GO enrichment analysis

The target genes of the selected miRNAs were predicted using the database miRDB, which collects miRNA-target interactions predicted based on support vector machines and high-throughput training datasets. All targets were downloaded from the website with *Homo sapiens* as the predicted species. To identify the regulatory functions of the miRNAs, Gene ontology (GO) annotation was performed for the target genes using ClueGO 30 in Cytoscape, which provided functional information on the gene products described with domain-specific ontologies.

### Transfection of hBMSCs during hepatic differentiation

Activators and inhibitors of nine selected miRNAs (hsa-miR-203a-3p, hsa-miR-204-5p, hsa-miR-101-3p, hsa-miR-26b-5p, hsa-miR-148a-3p, hsa-miR-93-3p, hsa-miR-423-3p, hsa-miR-222-3p, and hsa-miR-224-5p) were synthesized by GenePharma Co., Ltd. (Table S2). hBMSCs (third passage) were cultured in T25 flasks. When the cultured cells reached 70% confluence, a Targefect transfection kit 31 (Targeting Systems) was used to transfect the cells with 25 nM scrambled RNA, miRNA mimics (activators), or miRNA inhibitors. The transfection medium was changed to hepatic differentiation medium at 14 hours after transfection.

### Statistical analyses

The differential expression of miRNAs among undifferentiated hBMSCs (day 0), hBMSC-Heps at 10 and 20 days after hepatic differentiation, and PHHs was analyzed with the DESeq2 package in the R programming language. Significantly differentially expressed miRNAs were identified based on their logarithmic fold changes and P values. Unsupervised hierarchical clustering of the significantly differentially expressed miRNAs from the different groups was performed using the 'heatmap.2' function in the gplots package. This study employed the one-way analysis of variance (ANOVA) to analyze the significance in experiments contained more than two groups with the Tukey's test used for multiple comparisons. The p-value less than 0.05 was considered to indicate a significant difference. The results of the measurements are presented as the mean ± SD, unless otherwise noted.

## Results

### Phenotype and characteristics of hBMSC-Heps

**Figure 1:**
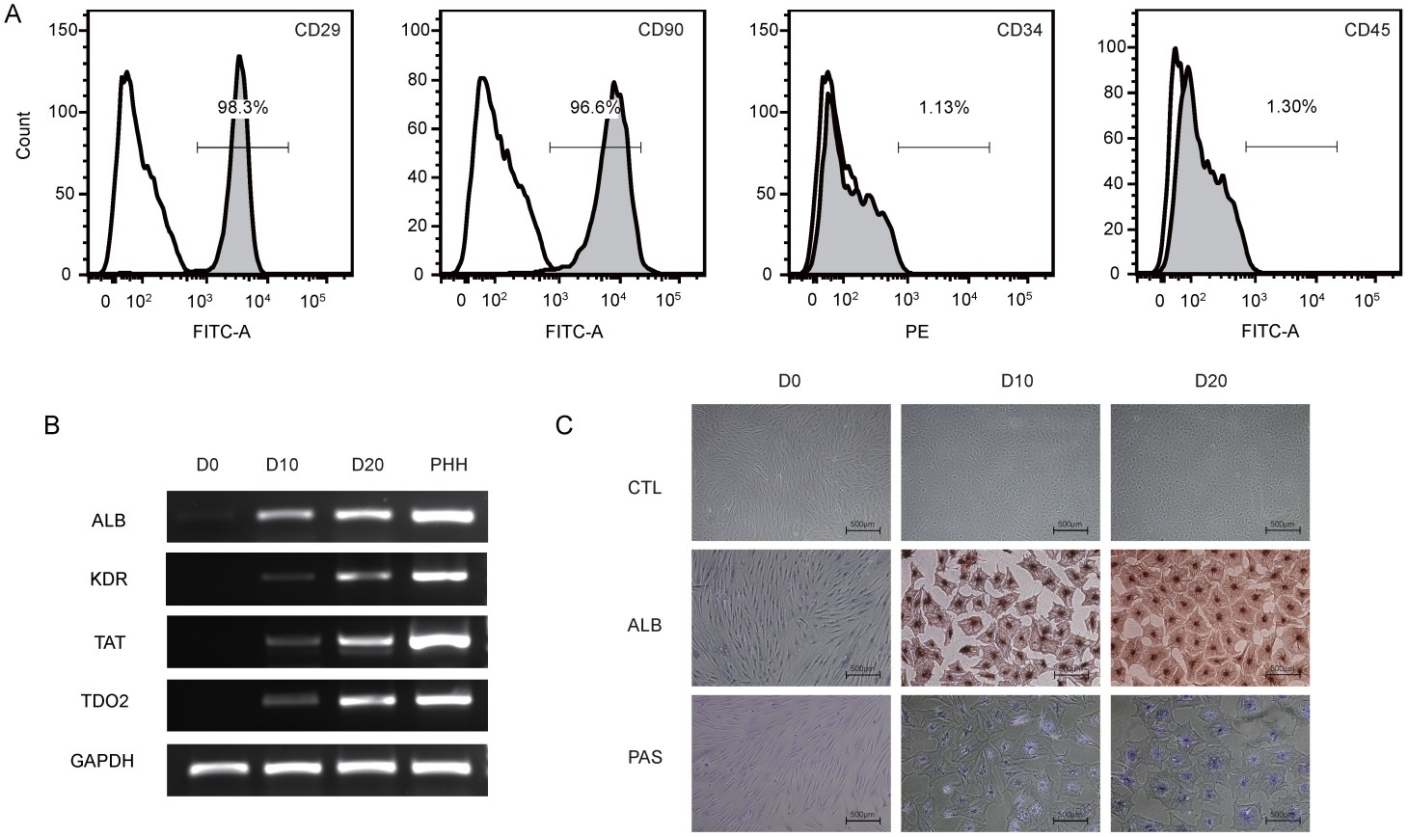
** (A)** Representative figures of flow cytometry analysis of CD29, CD90, CD34 and CD45. Third-passage hBMSCs were positive for CD29 (97.8±1.7%) and CD90 (96.3±1.7%) but negative for CD34 (2.5±1.6%) and CD45 (1.4±1.0%), n=3/group. **(B)** PCR analysis of the expression of ALB, KDR, TAT and TDO2 in hBMSCs (D0) and hepatic-differentiated hBMSCs (D10, D20). PHHs were used as positive controls. **(C)** Phase-contrast morphology and immunocytochemistry with ALB and PAS staining in undifferentiated hBMSCs (D0) and hBMSC-Heps (D10, D20); scale bar: 500 µm.

To identify the phenotype of the hBMSCs, third-passage hBMSCs were characterized by flow cytometry (Fig. 1A). The results showed that the hBMSCs used in this study were positive for CD29 (97.8±1.7%) and CD90 (96.3±1.7%) but negative for CD34 (2.5±1.6%) and CD45 (1.4±1.0%), suggesting that the cells used for further research possessed properties of typical hBMSCs. The phenotype of hBMSCs-Heps was determined by quantitative real-time polymerase chain reaction (qRT-PCR), immunocytochemistry and PAS staining. The qRT-PCR results for hepatocyte-specific genes showed that the expression levels of ALB, kinase insert domain receptor (KDR), tyrosine aminotransferase (TAT) and tryptophan 2,3-dioxygenase (TDO2) increased at D10 and reached higher levels at D20 after differentiation; undifferentiated hBMSCs were used as negative controls, and PHHs were used as positive controls (Fig. 1B). Under phase-contrast microscopy, hBMSC-Heps showed hepatocyte-like polygonal morphology with a low cytoplasm-to-nucleus ratio, whereas hBMSCs exhibited a fibroblast-like morphology. Immunocytochemistry and PAS staining showed that the hBMSCs-Heps were positive for ALB and glycogen at D10 and D20 (Fig. 1C); undifferentiated hBMSCs (D0) were used as negative controls. The above results indicated that the stem cells had typical hBMSC phenotypes, and after hepatic differentiation following an accepted protocol, the hBMSC-Heps showed hepatocyte-like cellular characteristics in terms of morphology and the expression of typical mature hepatocyte markers. To obtain the miRNA expression profiles of hBMSCs during different hepatic differentiation stages and in PHHs, miRNA sequencing was performed on undifferentiated hBMSCs (D0), hBMSC-Heps at D10 and D20, and PHHs. Based on the results of high-sensitivity sequencing, 1150 miRNAs were detected in at least one sample, and 837, 845, 861 and 813 miRNAs in miRBase v21 were identified in at least one sample of the D0 hBMSCs, D10 hBMSC-Heps, D20 hBMSC-Heps, and PHHs, respectively (Table S3).

### Qualitative analysis of differentially expressed miRNAs in hBMSC-Heps and PHHs

**Figure 2:**
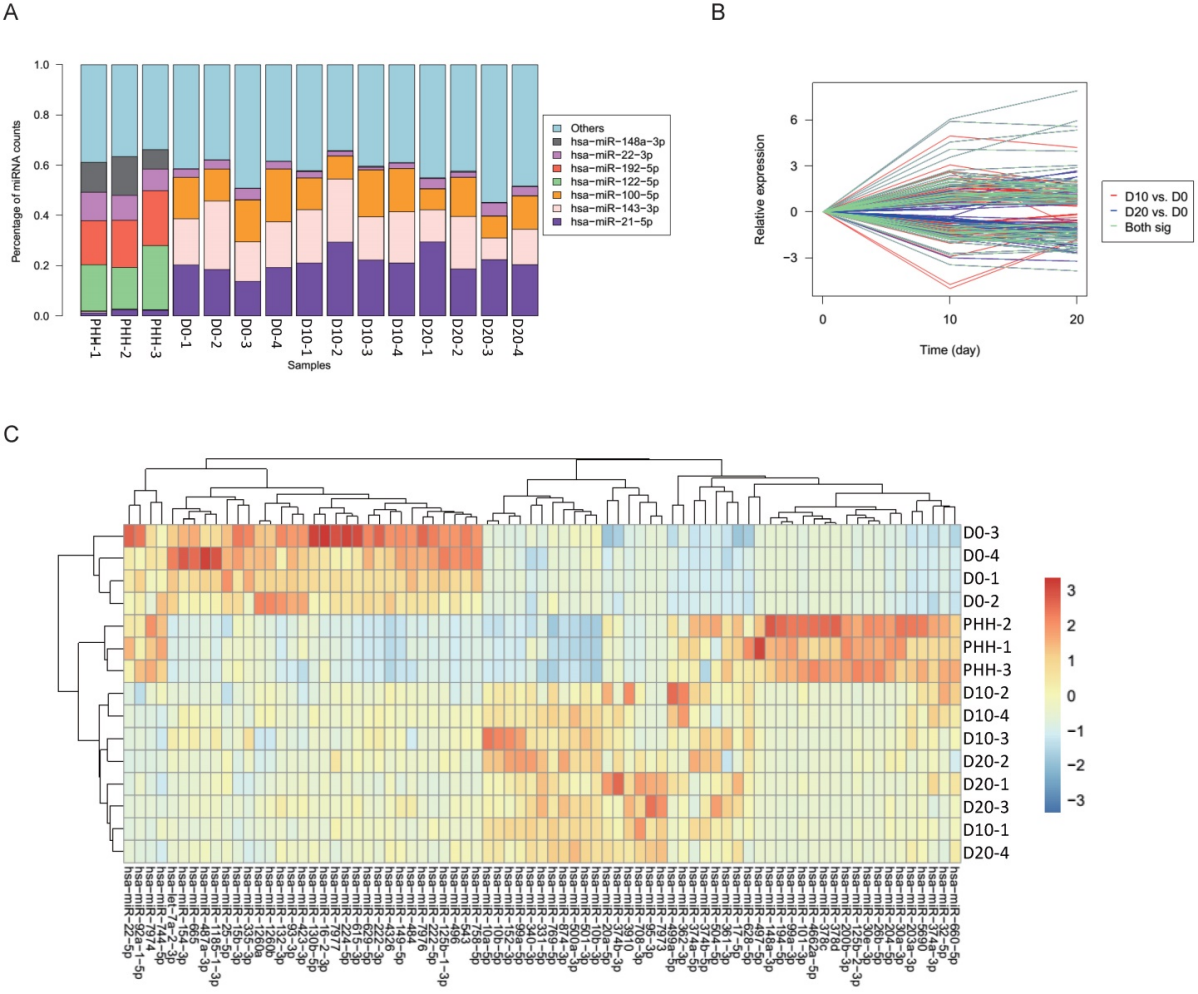
** (A)** Percentages of the top miRNAs in each sample as determined by sequencing. **(B)** Significantly differential expression of miRNAs between D10 and D0 is indicated with red lines, and that between D20 and D0 is indicated with blue lines; 77 of the miRNAs shared in both comparisons are indicated with green lines. **(C)** Clustering results of the samples and miRNAs using 77 differentially expressed miRNAs in undifferentiated hBMSCs (D0), hBMSC-Heps (D10 and D20) and PHHs.

**Figure 3:**
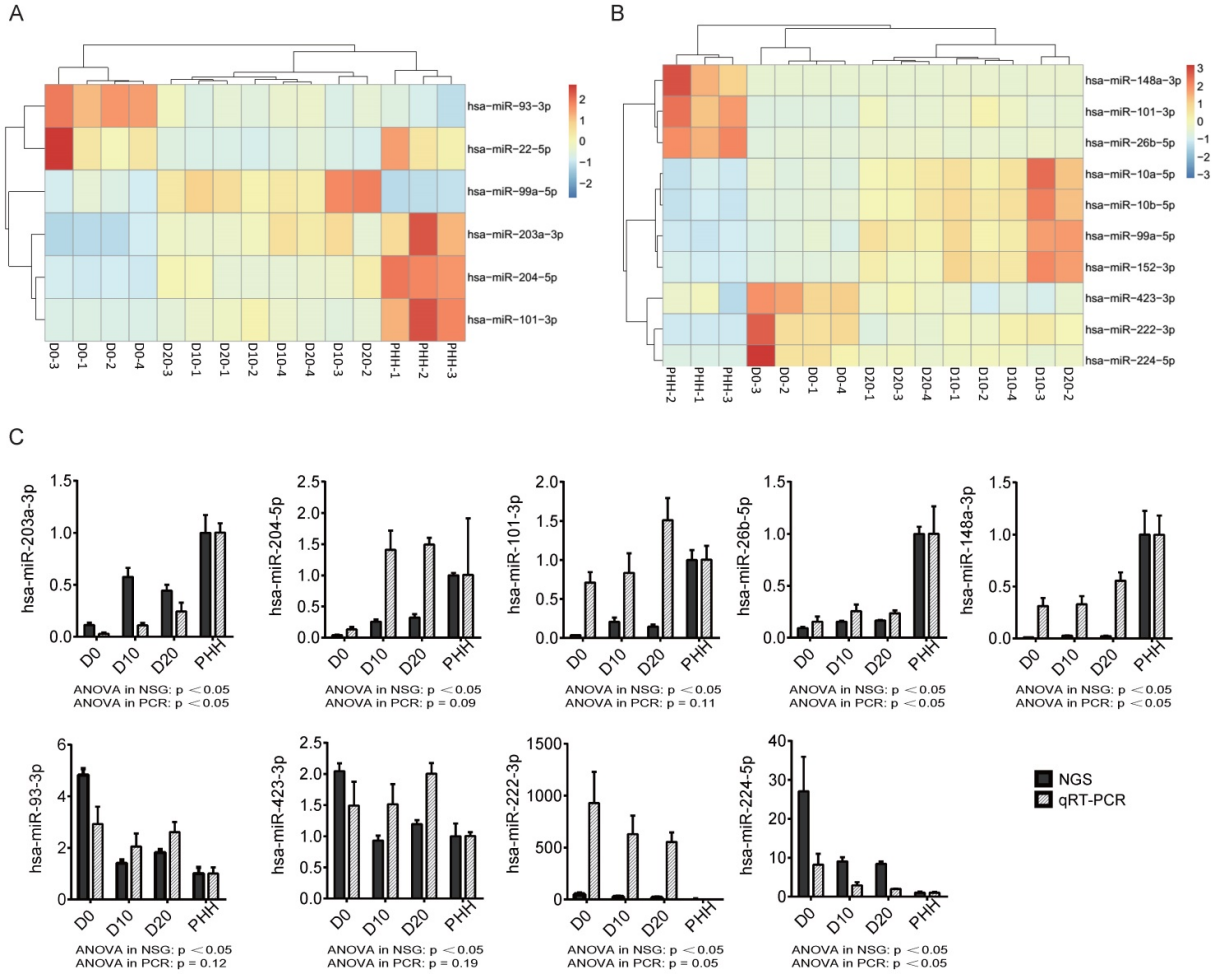
** (A)** Heatmap of 6 of the top 10 miRNAs significantly differentially expressed between D0 and D10/D20. **(B)** Heatmap of the top 10 most abundant miRNAs from the set of 77 differentially expressed miRNAs. **(C)** The changing trends of nine miRNAs in the four cell groups were measured by next-generation sequencing (NGS) and qRT-PCR. n=4 in D0/D10/D20 group, n=3 in HH group.

In all hBMSCs and their hepatic differentiation samples, hsa-miR-21-5p was the most abundant miRNA, with a high percentage that ranged from 13.7% to 29.4% of the total known miRNAs in each sample. However, hsa-miR-21-5p constituted only 1% or 2% of the miRNAs in the PHHs (Fig. 2A). The differential expression of miRNAs in hBMSC-Heps (D10 and D20) and undifferentiated hBMSCs (D0) was analyzed using DESeq2 v1.8.1. A set of 77 significantly differentially expressed miRNAs were obtained, of which 44 were upregulated and 33 were downregulated during hepatic differentiation (Fig. 2B). Clustering analysis based on the normalized expression of these 77 differentially expressed miRNAs revealed that all hBMSC-Heps were more similar to PHHs than undifferentiated hBMSCs (Fig. 2C). Among the 77 differentially expressed miRNAs, six miRNAs were in the top 10 significantly differentially expressed miRNAs when both the hBMSC-Hep groups (D10 and D20) were compared with the hBMSC group (Fig. 3A). Among the six miRNAs, four miRNAs that showed continuously increasing (hsa-miR-203a-3p, hsa-miR-204-5p, and hsa-miR-101-3p) or decreasing (hsa-miR-93-3p) trends from hBMSCs (D0) to hBMSC-Heps (D10 and D20) to PHHs were selected for subsequent testing. Furthermore, among the 77 differentially expressed miRNAs, the top 10 most abundant miRNAs in hBMSC-Heps at D20 were selected for additional clustering analysis (Fig. 3B). Three gradually upregulated miRNAs (hsa-miR-148a-3p, hsa-miR-101-3p, and hsa-miR-26b-5p) and another three downregulated miRNAs (hsa-miR-423-3p, hsa-miR-222-3p, and hsa-miR-224-5p) were selected for subsequent analysis. Overall, nine miRNAs showed gradually changing trends among the top 10 significantly differentially expressed and top 10 most abundant miRNAs were selected for validation.

### Validation of the hBMSC-Heps miRNA expression *in vitro* and *in vivo*

To validate the expression levels of the nine selected significantly differentially expressed miRNAs, we performed probe-based qRT-PCR *in vitro* with cultured hBMSC-Heps and *in vivo* with tissues from an hBMSC transplantation model in FHF pigs. The changing trends of the nine miRNAs in the four cell groups, as measured by sequencing, were confirmed by qRT-PCR analysis (Fig. 3C). The results showed that three gradually upregulated miRNAs (hsa-miR-203a-3p, hsa-miR-148a-3p, and hsa-miR-26b-5p) and another downregulated miRNA (hsa-miR-224-5p) were significantly changed (p<0.05).

**Figure 4:**
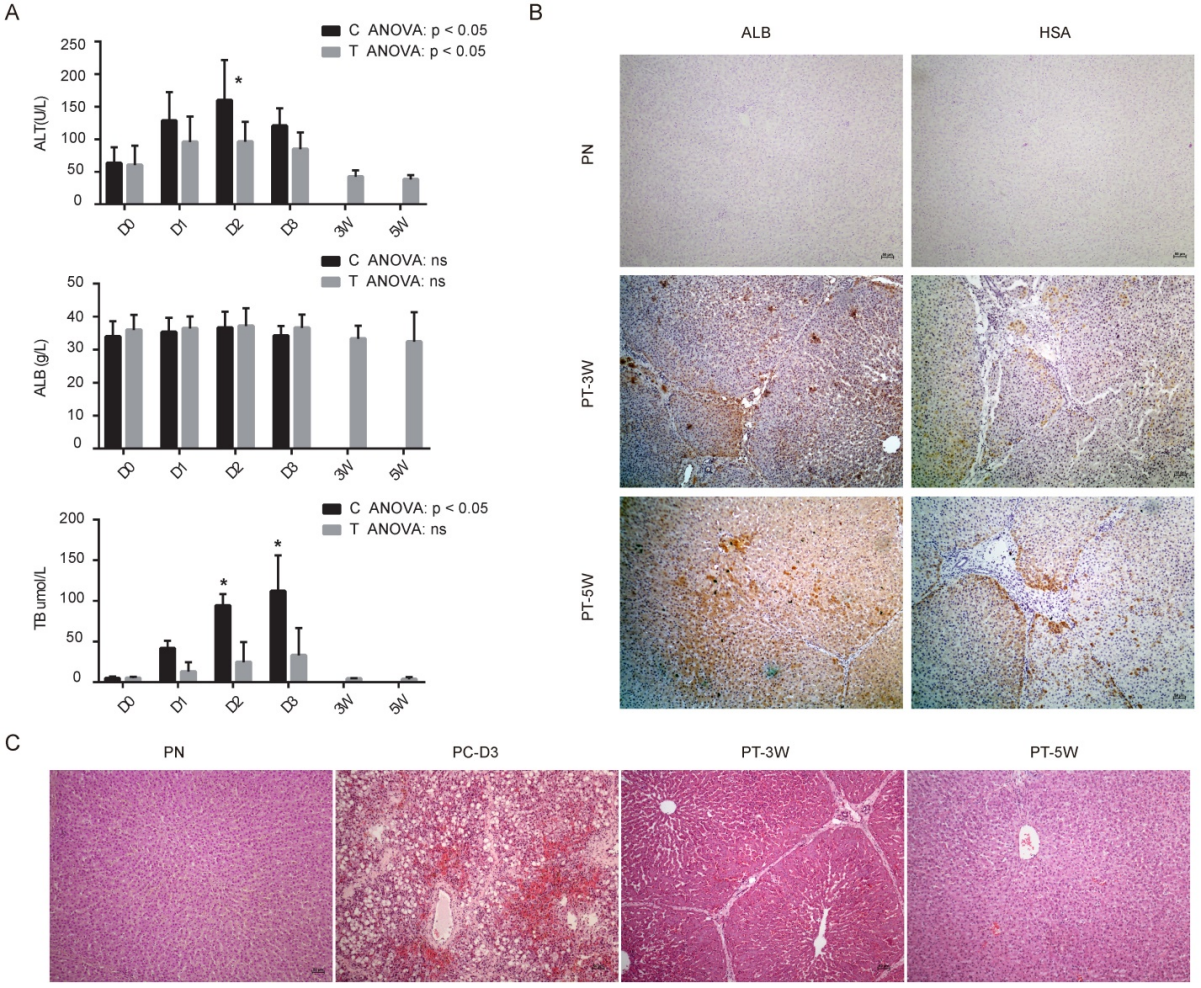
** (A)** Biochemical analysis of alanine aminotransferase (ALT), albumin (ALB) and total bilirubin (TB) in control group (C) and transplantation group (T), n=4/group. **(B)** Immunohistochemistry images of pig livers from the PN, PT-3W and PT-5W groups. Human ALB- and HSA-positive hepatocytes were widely distributed in the hepatic lobules in the PT-3W and PT-5W groups. Scale bar: 50 µm. **(C)** H&E staining of pig liver tissues in the PN, PC-D3, PT-3W and PT-5W groups; scale bar: 50 µm.

Our previous study revealed that implanted hBMSCs can quickly differentiate into hepatocyte-like cells *in vivo* and rescue FHF pigs 9. To characterize the role of miRNAs during the proliferation and differentiation of hBMSCs into hepatocyte-like cells *in vivo*, we validated the expression of the nine selected miRNAs in liver tissues using qRT-PCR. All five animals without transplantation died within 4 days, and 4 of 5 (80%) pigs survived beyond five weeks after transplantation. Biochemical analysis of Alanine aminotransferase (ALT), ALB and total bilirubin (TB) showed that the liver function of the transplantation group was much better than that of the control group (Fig. 4A). Analysis of the binding of human-specific antibodies (ALB^+^ and hepatocyte-specific antibody (HSA)^+^) by immunohistochemistry showed chimerization of human-derived hepatocyte-like cells in the pig liver tissues at weeks 3 and 5 (Fig. 4B) with normal pigs as normal control group (PN). H&E staining confirmed that pigs subjected to transplantation at week 3 (PT-3W) group and at week 5 (PT-5W) group showed repair of the damaged liver structure, whereas in FHF control group at day 3 after D-galactosamine induction (PC-D3) showed typical FHF histology with extensive hepatic necrosis and hemorrhage (Fig. 4C).

**Figure 5:**
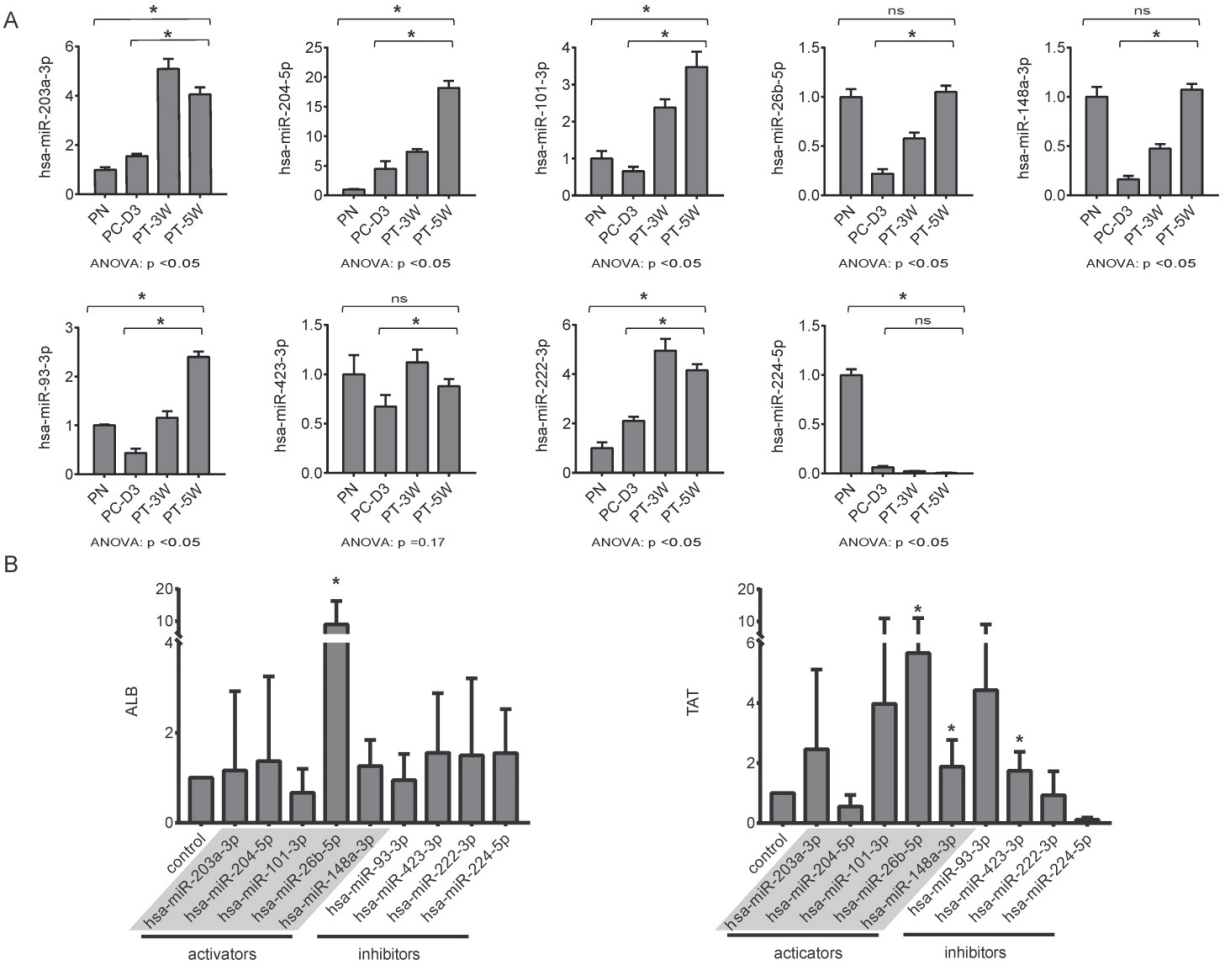
** (A)** The changing trends of nine miRNAs in the four liver tissue groups were measured by qRT-PCR, n=4/group. **(B)** hBMSCs were transfected with miRNA inhibitors or activators and subsequently analyzed for expression of the hepatic markers ALB and TAT by qRT-PCR, n=5/group. The control column was hBMSC-Heps from the standard differentiation protocol.

For qRT-PCR validation of the nine selected miRNAs, liver tissues were collected from pigs in the four groups, including two transplantation group (PT-3W, PT-5w) and the normal control PN group with the disease control PC-D3 group. The results of qRT-PCR analysis showed that the changing trends of the nine selected miRNAs in implanted hBMSCs during proliferation and differentiation *in vivo* were consistent with those of the miRNAs in cultured hBMSCs undergoing hepatic differentiation (Fig. 5A). The levels of hsa-miR-203a-3p, hsa-miR-204-5p, hsa-miR-101-3p, hsa-miR-26b-5p, hsa-miR-148a-3p hsa-miR-93-3p, hsa-miR-423-3p and hsa-miR-222-3p were much higher in the PT-5W group than in the PC-3D group (p<0.05) except of hsa-miR-224-5p, indicating that the expression of these miRNAs was induced by hBMSC transplantation. Furthermore, the expression of hsa-miR-26b-5p, hsa-miR-148a-3p and hsa-miR-423-3p showed no significant difference when compared with the PN group, indicating that the expression levels were restored to their baseline levels in the PT-5W group after hBMSCs transplantation.

### Functional characterization of the selected miRNAs during the differentiation of hBMSCs into hepatocyte-like cells with miRNA activators or miRNA inhibitors

**Figure 6:**
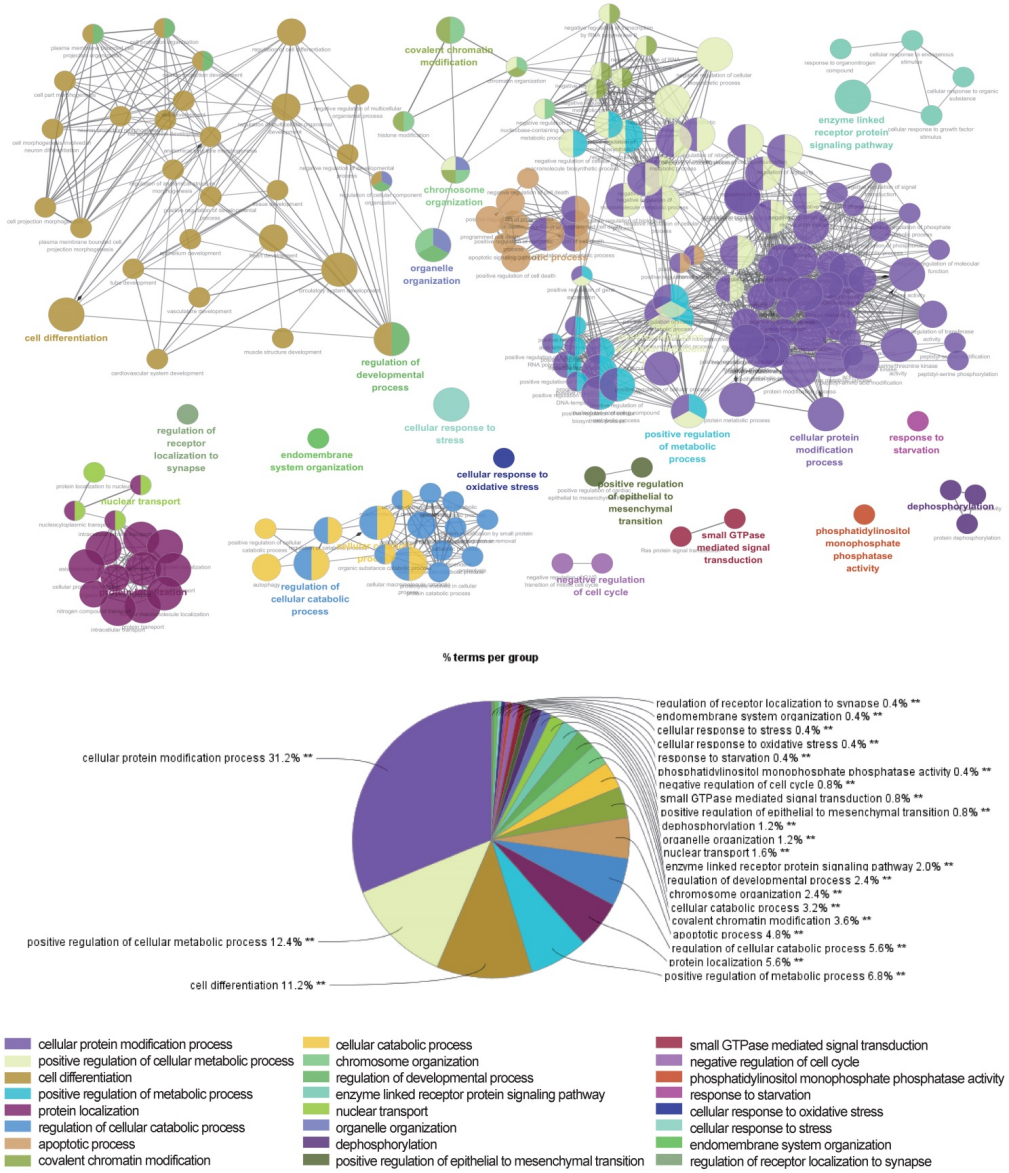
** Network of representative GO terms of 542 mRNAs.** Each node represents a GO term or a pathway. The colors of the node represent the GO functional groups with the group names showed in the list below the network. The terms are connected based on shared genes.

The above data suggested that the selected miRNAs are critical regulators during hepatic differentiation. To clarify the roles of these nine miRNAs, we transfected hBMSCs with miRNA activators or miRNA inhibitors for functional studies. Improvement in hepatic differentiation was indicated by the expression of the hepatocyte-specific markers ALB and TAT. Overexpression of hsa-miR-26b-5p and hsa-miR-148a-3p, as well as inhibition of hsa-miR-423-3p, showed the capacity to enhance hepatic differentiation, as indicated by increased ALB and TAT expression in the transfected cells (Fig. 5B). The function of a miRNA is ultimately performed by the target mRNAs whose expression it regulates. To gain a better understanding of the role of miRNAs in the proliferation and differentiation of hBMSCs, we searched for the targets of the differentially expressed miRNAs in miRDB. For three functional miRNAs (hsa-miR-26b-5p, hsa-miR-148a-3p and hsa-miR-423-3p), 1044 targets were obtained from miRDB. There were 542 liver specific mRNAs among the 1044 targets existed in the data sets of human hepatocytes. To clarify the functions of these 542 mRNAs, clustering analysis of their expression patterns was performed. GO enrichment analysis was used to systematically describe the relationships among the mRNAs and their possible biological mechanisms. Ultimately, 250 GO terms were identified and clustered into 24 GO functional groups (Fig. 6). The main terms with significant enrichment among the genes were related to the characteristics of stem cells, such as cell differentiation, developmental process, and several processes were related to hepatocyte functions, such as protein modification process, metabolic process and catabolic process.

## Discussion

Stem cell-based strategies have been the most promising approach for patients suffering from end-stage liver disease and FHF. The *in vitro* hepatic differentiation process of hBMSCs has been characterized in many studies from our group and others. However, the regulatory mechanism, the efficacy of hBMSC-Heps compared with hepatocytes, and the risk of *in vivo* application remain unclear.

Mesenchymal stem cells have multipotent differentiation capacity; they can differentiate into multiple types of cells under specific circumstances. In addition to analysis of phenotypic changes, several classic biochemical molecules (e.g., ALB, TAT and TDO2) have been used as biomarkers to evaluate hBMSC-Heps. Previous studies have revealed that each type of stem cells differentiation can be represented by specific miRNA markers 25. However, the transcriptional and translational expression patterns that hBMSCs present during different developmental stages and the factors that determine the differentiation pattern changes remain unclear. Our results of miRNA profiling at the molecular level revealed the activation of signaling pathways during stem cells hepatic differentiation. The results also indicated the extent to which stem cells differentiate into hBMSC-Heps and enabled comprehensive functional characterization of hBMSC-Heps compared to PHHs. MicroRNA-148a has been reported as a key molecule during liver development, as its expression is higher in the fetal liver than in the adult liver. In this study, the expression of hsa-miR-148a-3p rapidly increased during the hepatic differentiation of hBMSCs, but the level was still lower than its expression in PHHs. Furthermore, the most abundantly expressed miRNA during stem cells hepatic differentiation, hsa-miR-21-5p, played an important role in hepatic proliferation, but its expression remained high in hBMSC-Heps. In contrast, the liver-specific miRNA hsa-miR-122-5p, which accounted for ~20% of all expressed miRNAs in PHHs, did not accumulate to appreciable levels in hBMSC-Heps. These results showed that the differentiation of stem cells *in vitro* was still not complete.

*In vivo*, stem cells or progenitor cells contribute to supplying hepatocytes for the maintenance of liver homeostasis and for recovery from injury. In this study, the expression levels of nine selected miRNAs were tracked by qRT-PCR in liver tissues from FHF pigs receiving hBMSC transplantation. The levels of the functional miRNAs indicated a significant difference in hepatic regeneration function in the PT-3W group compared to the PC-3D group; the levels returned to baseline in the PT-5W group and were similar to the levels in the PN group. Our confirmation of the miRNA sequences and the qRT-PCR results demonstrated that the selected miRNAs could be important regulators during stem cells transplantation. The above results imply that the current protocols for hepatic differentiation of hBMSCs successfully provide hBMSC-derived functional hepatocyte-like cells but still need substantial improvements. Understanding the hepatic differentiation of hBMSCs *in vivo* yields insight into molecules that can be used to instruct stem cells to differentiate and grow.

The function of miRNAs in controlling hepatocyte proliferation during liver regeneration and in improving the differentiation of hepatic precursor cells has been reported 31, 32, 33, but the mechanism needs to be elucidated 34. In this study, miRNA activators and miRNA inhibitors were used for gain-of-function or loss-of-function studies. The results showed that hsa-miR-26b-5p, hsa-miR-148a-3p and hsa-miR-423-3p played key roles in hBMSC hepatic differentiation and promoted hepatic gene expression during differentiation. MicroRNA-148a has been reported to be a regulator of the low-density lipoprotein (LDL) receptor 35 and can promote hepatic lipid metabolism in mice 36; it is also one major member of a set of six miRNAs mediating the induction of human umbilical cord lining-derived mesenchymal stem cell (MSC) differentiation into hepatocyte-like cells. Other studies have indicated that MicroRNA-26b participates in regulating the chemotactic response of MSCs toward HGF 37 and regulates proliferation, angiogenesis and apoptosis in hepatocellular carcinoma 38. Furthermore, recent studies have determined the role and mechanism of miR-423-5p in hepatic glucose and lipid metabolism 39. These results indicate that hsa-miR-26b-5p, hsa-miR-148a-3p and hsa-miR-423-3p could play an essential role in hepatic differentiation of hBMSCs. While a single mRNA can be regulated by several miRNAs, it is difficult to elevate hepatic function completely by a single miRNA, so the combined manipulation of miRNAs provides a new perspective to enhance stem cells differentiation. Considering the functions of miRNAs in inducing the proliferation of mature hepatocytes and improving the differentiation of hBMSCs, miRNA-based technologies provide attractive tools for improving therapeutic approaches involving hBMSCs.

## Conclusions

Our study identified the miRNA profile of hBMSCs in several hepatic differentiation states and validated the miRNA basis of hBMSC-Heps *in vitro* and *in vivo* through hBMSC transplantation into FHF pigs. Due to the powerful ability of miRNAs to fine-tune biological processes relevant to hepatic differentiation, developmental growth and cell functions, our discovery of specific miRNAs may enable significant improvements in the evaluation and acceleration of hBMSC differentiation into hepatocytes and may lead to beneficial clinical applications in the future.

## Supplementary Material

Supplementary tables.

## Abbreviations

ALB: albumin
ALT: alanine aminotransferase
FHF: fulminant hepatic failure
GO: Gene ontology
hBMSCs: human bone marrow mesenchymal stem cells
hBMSC-Heps: hBMSC-differentiated hepatocyte-like cells
hHGF: human hepatocyte growth factor
HSA: hepatocyte-specific antibody
H&E: Hematoxylin and eosin
ITS: insulin, transferrin, and selenium
miRNAs: microRNAs
OSM: oncostatin M
PAS: periodic acid-Schiff
PHHs: primary human hepatocytes
qRT-PCR: quantitative real-time polymerase chain reaction
TAT: tyrosine aminotransferase
TB: total bilirubin
TDO2: tryptophan 2,3-dioxygenase
