# Supplementary material for: MicroRNA Profile of Human Bone Marrow Mesenchymal Stem Cells during Hepatic Differentiation and Therapy
Source: Int J Med Sci. 2022 Jan 1;19(1):152–63. doi: 10.7150/ijms.67639 (PMC8692113; doi:10.7150/ijms.67639)
Supplement: Supplementary file 1 — Supplementary tables. [file ijmsv19p0152s1.pdf]

Supplementary tables

Supplementary table 1. Primer sequences of liver-specific genes. NCBI: National Center for Biotechnology Information.

Gene	NCBI accession number	Product size	Forward	Reverse
ALB	NM_000477.5	166	TGGCACAATGAAG TGGGTAA	CTGAGCAAAGGCA ATCAACA
KDR	NM_002253.3	218	GTGACCAACATGG AGTCGTG	TGCTTCACAGAAG ACCATGC
TAT	NM_000353.1	236	AGCCATTGTGGAC AACATGA	TAGCTTCTAGGGG TGCCTCA
TDO2	NM_005651.3	222	GGGAACTACCTGC ATTTGGA	GTGCATCCGAGAA ACAACCT
GAPDH	NM_002046.3	113	CTCTCTGCTCCTCC TGTTCG	ACGACCAAATCCG TTGACTC

Supplementary table 2. Nine miRNAs selected for validation.

**miRNA name**	**Sequence**	**Functional study reagent type**
**hsa-miR-203a-3p**	GUGAAAUGUUUAGGACCACUAG	mimic
**hsa-miR-204-5p**	UUCCCUUUGUCAUCCUAUGCCU	mimic
**hsa-miR-101-3p**	UACAGUACUGUGAUAACUGAA	mimic
**hsa-miR-26b-5p**	UUCAAGUAAUUCAGGAUAGGU	mimic
**hsa-miR-148a-3p**	UCAGUGCACUACAGAACUUUGU	mimic
**hsa-miR-93-3p**	ACUGCUGAGCUAGCACUUCCCG	inhibitor
**hsa-miR-423-3p**	AGCUCGGUCUGAGGCCCCUCAGU	inhibitor
**hsa-miR-222-3p**	AGCUACAUCUGGCUACUGGGU	inhibitor
**hsa-miR-224-5p**	CAAGUCACUAGUGGUUCCGUU	inhibitor

Supplementary table 3. Summary of miRNA sequence data for each sample.

Sample	Total reads	Reads mapped	% mapped	Known miRNA number	Known miRNA number
**Total:**	135352192	108225612	80%	1150	1150
**PHH-1**	7032824	5005090	71%	678	813
**PHH-2**	5940753	4624146	78%	589	
**PHH-3**	9883680	8183918	83%	674	
**D0-1**	11750774	8636277	74%	693	837
**D0-2**	11750135	9915760	84%	712	
**D0-3**	5781418	4447322	77%	621	
**D0-4**	6211768	4478916	72%	602	
**D10-1**	8323432	7710094	93%	667	845
**D10-2**	7384872	4530274	61%	596	
**D10-3**	15322431	14692364	96%	766	
**D10-4**	7583614	5876665	78%	600	
**D20-1**	17937170	13510503	75%	781	861
**D20-2**	6488875	6063218	93%	635	
**D20-3**	6214654	4761530	77%	661	
**D20-4**	7745792	5789535	75%	640	
